# CXXC finger protein 4 inhibits the CDK18‐ERK1/2 axis to suppress the immune escape of gastric cancer cells with involvement of ELK1/MIR100HG pathway

**DOI:** 10.1111/jcmm.15625

**Published:** 2020-07-26

**Authors:** Ping Li, Dongfang Ge, Pengfei Li, Fangyong Hu, Junfeng Chu, Xiaojun Chen, Wenbo Song, Ali Wang, Guangyu Tian, Xiang Gu

**Affiliations:** ^1^ Department of Central Laboratory Huaian Tumor Hospital & Huaian Hospital of Huaian City Huaian China; ^2^ Department of General Surgery Huaian Tumor Hospital & Huaian Hospital of Huaian City Huaian China; ^3^ Department of Experimental Surgery‐Cancer Metastasis Medical Faculty Mannheim Ruprecht Karls University Mannheim Germany; ^4^ Department of Oncology Jiangdu People's Hospital Affiliated to Medical College of Yangzhou University Yangzhou China

**Keywords:** CXXC finger protein 4, ETS domain‐containing protein‐1, gastric cancer, immune escape, long non‐coding RNA MIR100HG

## Abstract

Gastric cancer, is the fourth most common tumour type yet, ranks second in terms of the prevalence of cancer‐related deaths worldwide. CXXC finger protein 4 (CXXC4) has been considered as a novel cancer suppressive factor, including gastric cancer. This study attempted to investigate the possible function of CXXC4 in gastric cancer and the underlying mechanism. The binding of the ETS domain‐containing protein‐1 (ELK1) to the long non‐coding RNA MIR100HG promoter region was identified. Then, their expression patterns in gastric cancer tissues and cells (SGC7901) were detected. A CCK‐8 assay was used to detect SGC7901 cell proliferation. Subsequently, SGC7901 cells were co‐cultured with CD3+ T cells, followed by measurement of CD3+ T cell proliferation, magnitude of IFN‐γ+ T cell population and IFN‐γ secretion. A nude mouse model was subsequently developed for in vivo validation of the in vitro results. Low CXXC4 expression was found in SGC7901 cells. Nuclear entry of ELK1 can be inhibited by suppression of the extent of ELK1 phosphorylation. Furthermore, ELK1 is able to bind the MIR100HG promoter. Overexpression of CXXC4 resulted in weakened binding of ELK1 to the MIR100HG promoter, leading to a reduced proliferative potential of SGC7901 cells, and an increase in IFN‐γ secretion from CD3+ T cells. Moreover, in vivo experiments revealed that CXXC4 inhibited immune escape of gastric cancer cells through the ERK1/2 axis. Inhibition of the CXXC4/ELK1/MIR100HG pathway suppressed the immune escape of gastric cancer cells, highlighting a possible therapeutic target for the treatment of gastric cancer.

## 1 INTRODUCTION

Gastric cancer is currently the second most common cause of cancer‐related deaths in China and worldwide, while being also the second most commonly diagnosed type of cancer.^1^, ^2^ Interestingly, gastric cancer can be treated with relative ease if caught at an early stage. However, patients with gastric cancer at an advanced stage show extensive invasion and metastasis beyond the stomach leading to many difficulties in treatment and a generally poor prognosis.^3^ Lack of accurate molecular markers for early‐stage gastric cancer can lead to progression of this disease in up to 70% of patients.^3^ Generally, the treatment for gastric cancer contains standard gastrectomy as well as neoadjuvant chemotherapy, radiotherapy and molecular‐targeted therapies.^3^ Furthermore, according to the description of tumour status (T/N/M and stage), combined treatments which relieve suffering and acquire best results are performed.^3^ Immunotherapy is a novel treatment option using immune tumour vaccines or antibodies against tumour antigens to stimulate one's immune system to fight the tumour. Immune checkpoint inhibitors such as programmed death 1/programmed cell death‐ligand 1 (PD‐1/PD‐L1) are now widely used as targets for immunotherapy, as they activate effector T cells to kill stomach cancer cells.^4^

CXXC proteins are a group of zinc finger proteins that preferentially bind to CpG islands on genomic DNA thus regulating transcription.^5^ CXXC4 is a tumour suppressor expressed at low levels in tumours compared to normal tissues. Thus, very low expression of CXXC4 correlates with poor survival outcome.^6^ An intriguing finding is that down‐regulation of CXXC4 can activate the Wnt pathway, which leads to malignant renal cell carcinoma.^6^ Thus, CXXC4 functions as a tumour suppressor in gastric cancer, and can be negatively controlled by enhancer of zeste homolog 2 and participates in mitogen‐activated protein kinase (MAPK) axis to suppress tumour growth.^6^, ^7^ More importantly, CXXC4 has been found to inhibit the extent of ETS domain‐containing protein‐1 (ELK1) phosphorylation,^8^ which is relevant as phosphorylation of ELK1 promotes the development of gastric cancer.^9^ ELK1 is a transcription factor binding to purine‐rich DNA sequences and has been shown to be up‐regulated in various cancers.^10^ ELK1 may contribute to CD8+ T cell differentiation by activating the extracellular regulated protein kinases (ERK); this may help eradicate cancer cells by activating T cells.^11^ However, the relationship between CXXC4 and ELK1 in gastric cancer is not clear. In this study, we conducted a series of bioinformatic analyses to explore potential genes involved in gastric cancer. Based on the microarray dataset of gastric cancer GSE49051 in the GEO database, we successfully isolated CXXC4 as a significantly down‐regulated gene in gastric cancer, while the StarBase database revealed a significant up‐regulation of ELK1 in gastric cancer. Interestingly, we found CXXC4 and ELK1 were co‐expressed in gastric cancer using the Chipbase website. Furthermore, Chipbase also revealed that ELK1 and MIR100HG are also co‐expressed, and, at the same time, ELK1 binding sites in MIR100HG promoter region were predicted by JASPAR. MIR100HG is a long non‐coding RNA (lncRNA) reported to be an oncogene in multiple cancers including colorectal, laryngeal squamous cell carcinoma and triple‐negative breast cancer.^12^, ^13^, ^14^ In gastric cancer, high expression of MIR100HG is positively correlated with clinical stage, tumour invasion and distal metastasis, thus potentially serving a potential prognostic biomarker for gastric cancer.^15^ However, how these molecules can play a role in tumour immunotherapy remains unclear. Based on reported studies and bioinformatic analyses, we have been suggested the potential role of CXXC4/ELK1/MIR100HG in gastric cancer.

In our investigation, we unravelled the function of CXXC4 in T cell stimulation. CXXC4 inhibited the phosphorylation of ELK1 reducing its nuclear translocation, which restrained the expression of MIR100HG. Finally, the signalling of cyclin‐dependent kinase 18‐extracellular signal‐regulated kinases1/2 (CDK18‐ERK1/2) was prohibited and activated T cells reduced the proliferation of gastric cancer cells.

## 2 MATERIALS AND METHODS

### 2.1 Ethics statement

Our experiments were carried out with the approval of the Ethics Committee of Huaian Tumor Hospital, which also complied with the principles of the Declaration of Helsinki. Signed informed consents were provided by all participants. Moreover, all animal experiments were employed under the approval of the Institutional Animal Care and Use Committee of Huaian Tumor Hospital.

### 2.2 Sample collection

Eighty‐six resected gastric cancer specimens and adjacent normal specimens (>5 cm) were obtained from the patients (56 males and 30 females, aged 34.41‐65.61 years) of Huaian Tumor Hospital during December 2016 to January 2018. All patients received no chemotherapy or radiotherapy before operation. All pathological specimens were subjected to neutral formalin fixation and paraffin embedding. Histologic diagnosis and grading refer to World Health Organization Classification of Tumours of the Digestive System.

### 2.3 Cell treatment

Normal gastric epithelial cell line (GES‐1) (CBP60512) and gastric cancer cells SGC7901 (CBP660500), BGC‐823 (CBP60477), SNU‐1 (CBP660501) and HGC‐27 (CBP60480) were provided by COBIOER Biosciences Co., Ltd. Then, 10% foetal bovine serum (FBS)‐containing Dulbecco's modified eagle's medium (for GES‐1 and HGC‐27 cells) and 10% FBS‐containing Roswell Park Memorial Institute (RPMI) 1640 medium (for SGC7901, BGC‐823 and SNU‐1 cells) were used to culture the cells.

Under the manufacturer's instructions, pGCSIL‐PUR lentivirus of encoding shRNA against CXXC4 (sh‐CXXC4) and Lenti‐OE lentivirus of overexpressing CXXC4, MIR100HG or CDK18 from Shanghai Genechem Co., Ltd., as well as ERK1/2 pathway inhibitor GDC‐0994 from MedChemExpress (10 µmol/L, HY‐15947), were used for cell transfection. After 6 hours, the culture medium was renewed and the cells were further cultured for 48 hours, followed by subsequent experiments.

### 2.4 Cell proliferative potential detection

After overnight culture in a 96‐well plate (10^4^ cells per well), the cells were transfected and cultured for 0, 1, 2 and 3 days. Then, 10 µL of the cell counting kit 8 (CCK‐8) solution was added for 2 hours of incubation. The absorbance value of each well at 450 nm was measured for plotting a growth curve.^16^, ^17^

### 2.5 Isolation and identification of T cells

The heparin anticoagulant was put into a centrifuge, and the serum was discarded. Red blood cell lysis buffer was used for 10 minutes to lyse haemocytes at room temperature. After 5‐minute centrifugation and supernatant removal, the cell pellets were washed and resuspended in 5 mL sterile phosphate buffer saline (PBS) solution, followed by cell counting. After that, the cells positive for PE‐CD3+ (130‐113‐129; 1:50; Miltenyi biotec) were selected using flow cytometry.

### 2.6 In vitro cell co‐culture

The CD3+ T cells (10^5^ cells/96‐well plate) labelled by 5(6)‐Carboxyfluorescein diacetate succinimidyl ester (CFSE) (S1076; Beijing Solabio Life Sciences Co., Ltd.) were co‐cultured with SGC7901 cells of the different transfection groups for 48 hours in medium containing recombinant human Interleukin‐2 (rhIL‐2) (20 IU/mL), anti‐CD3 (2 μg/mL) and anti‐CD28 (1 μg/mL). Subsequently, flow cytometry was employed.^18^

### 2.7 Proliferation detection of T cells co‐cultured with gastric cancer cells

A 96‐well plate was coated with anti‐CD3/anti‐CD28 tetramer antibodies (STEMCELL Technologies Inc). IL‐2 (20 IU/mL) was added to the collected CD3+ T cells. The CD3+ T cells labelled by CFSE were co‐cultured with gastric cancer cells in the RPMI 1640 medium at 37°C with 5% CO_2_. The content difference of CFSE was measured by flow cytometry, and cells with low CFSE signal were considered to be proliferating cells.^19^

### 2.8 Flow cytometry

The cells were prepared into a single cell suspension and then resuspended in the staining buff (BD Biosciences Inc). T cells were added with PerCP‐CD3 (#100326; Armenian Hamster; 1:100; BioLegend) and Pacific blue‐interferon‐γ (IFN‐γ; #505817, Rat; 1:50; BioLegend), and Pacific blue‐IFN‐γ was added after immobilization and permeabilization. The cells were measured by BD fluorescent‐activated cell sorting canto Ⅱ (BD immunology systems, BD Biosciences Inc) and analysed by flow Jo software.^20^, ^21^

### 2.9 Enzyme‐Linked Immunosorbent Assay (ELISA)

The cytokine IFN‐γ secreted by T cells was determined by ELISA. In short, IFN‐γ specific antibody and cells were incubated in a 96‐well plate at 4°C for 24 hours. Then, the cells were washed with PBS and sealed with blocking buffer. A total of 100 mL detection antibody was added into the plate and then 100 μL of diluted streptavidin peroxidase. The reaction was stopped by adding 3,3′,5,5′‐Tetramethylbenzidine substrate and sulphuric acid. Enzyme activity was measured as optical density value at the wavelength of 450 nm by using ELISA reader.^22^

### 2.10 Dual‐luciferase reporter gene assay

The gastric cancer gene microarray GSE49051 was obtained from the Gene Expression Omnibus database (https://www.ncbi.nlm.nih.gov/geo/), which contained 3 normal samples and 3 cancer samples. The limma package was used for screening differentially expressed genes (DEGs), with |log of fold change| > 1.0, adj.*P*.Val < .05 as the screening criteria. The expression of gastric cancer‐related factors in The Cancer Genome Atlas was assessed by StarBase database (http://starbase.sysu.edu.cn/). The related mechanism was predicted by searching the co‐expression relationship between CXXC4 and ELK1 on Chipbase website (http://rna.sysu.edu.cn/chipbase/). The possible binding sites of ELK1 protein in MIR100HG promoter were analysed by UCSC (http://genome.ucsc.edu/) and JASPAR (http://jaspar.genereg.net/). The recombinant luciferase reporter gene vector of truncated or mutated binding site and ELK1 expression vector were constructed and cotransfected into SGC7901 cells for the dual‐luciferase reporter gene assay. The cotransfection of negative control for overexpression (oe‐NC) and oe‐ELK1 with 2Kb luciferase reporter plasmids in MIR100HG promoter region was carried out to assess whether ELK1 could bind to the MIR100HG promoter region. The expression vector pRL‐TK (Takara Biotechnology Co., Ltd.) of Renilla luciferase was used as the internal reference. After 48 hours of transfection, the cells were collected and lysed under the instruction of the luciferase detection kit (k801‐200; BioVision Technologies Inc). The luciferase reporter gene was analysed using a dual‐luciferase reporter gene analysis system (Promega Corporation). Relative light unit (RLU) was determined by a fluorometer with Renilla luciferase taken as the internal reference. The luciferase activity was expressed by the ratio of firefly RLU to renilla RLU.

### 2.11 Chromatin immunoprecipitation (ChIP)

The cells were fixed and cross‐linked with 1% formaldehyde for 10 minutes. Then, the chromatin was broken by ultrasonic wave and centrifuged at 12 000 *g* for 10 minutes at 4°C. The supernatant was collected and divided into two tubes then incubated with antibody to immunoglobulin G (IgG) (ab109489; 1:300; Abcam Inc) for NC and the specific antibody to phosphorylated ELK1 (p‐ELK1) (ab28818; 1:100; Abcam) at 4°C overnight. Protein Agarose/Sepharose was used to precipitate DNA protein complex. After centrifugation for 5 minutes at 12 000 *g*, the supernatant was discarded and the non‐specific complex was washed. De‐crosslinking was conducted at 65°C overnight, and the phenol/chloroform was used to extract, purify and collect DNA fragments. The binding of ELK1 protein to MIR100HG promoter was assessed by RT‐qPCR.

### 2.12 Subcellular fraction

The cells were resuspended with Hypotonic buffer A [10 mmol/L N‐2‐hydroxyethylpiperazine‐N‐ethane‐sulphonicacid (HEPES) (pH = 7.5), 0.5 mmol/L Dithiothreitol (DTT), 10 mmol/L KCl, 1.5 mmol/L MgCl_2_] containing protease and RNase inhibitors (n8080119; Thermo Fisher Scientific). After incubation on ice for 10 minutes, the cells were centrifuged at 1000 *g* for 10 minutes at 4°C. The supernatant was further centrifuged at 15 000 *g* for 15 minutes to obtain the cytoplasm. The precipitate was washed twice with hypotonic buffer and resuspended with Hypotonic buffer B [10 mmol/L HEPES (pH = 7.5), 10 mmol/L KCl, 1.5 mmol/L MgCl_2_, 0.5 mmol/L DTT, 0.5% Nonidet P‐40]. After incubation at 4℃ for 30 minutes, the precipitate was centrifuged at 4°C at 6000 × g for 10 minutes and washed with hypotonic buffer. Then, the precipitate was resuspended with Radio Immunoprecipitation Assay buffer (50 mmol/L Tris HCl [pH = 7.5], 1500 mmol/L KCl, 1% Nonidet P‐40, 0.5% sodium deoxycholate, 0.1% sodium dodecyl sulphate, 1 mmol/L ethylenediaminetetraacetic acid pH = 8.0) containing protease inhibitor and RNase inhibitor. After incubation at 4°C for 30 min, the precipitate was centrifuged at 15 000 *g* for 20 minutes, and the collected supernatant contained the nuclei.

### 2.13 RNA isolation and quantitation

Reverse transcription quantitative polymerase chain reaction (RT‐qPCR) was carried out under the instructions of the TaqMan Gene Expression Assays protocol (Applied Biosystems, Thermo Fisher Scientific), and glyceraldehyde‐3‐phosphate dehydrogenase (GAPDH) was used as the internal reference (Table 1). The relative expression of each target gene was calculated by 2^−ΔΔCt^ method.^23^, ^24^

**Table 1:** Primer sequences for RT‐qPCR

	Forward primer (5′‐3′)	Reverse primer (5′‐3′)
CXXC4	CTCATCAACTGTGGCGTCTG	TTAGTTTGCCCTTCATTTCC
MIR100HG	CCCAGTGCAAGGACAAAGA	GCAGAGGAGGTGTCTTCAGG
CDK18	TTCTCCCAACAGACAGCGG	GCAGCTGGAACATGAAAATCTTG
β‐actin	CACTGTGCCCATCTACGAGG	TAATGTCACGCACGATTTCC

Abbreviations: CDK18, cyclin‐dependent kinase 18; CXXC4, CXXC finger protein 4; RT‐qPCR, reverse transcription quantitative polymerase chain reaction.

#### 2.13.1 Western blot analysis

Total protein from tissues or cells was extracted by adding phenylmethylsulfonyl fluoride and protease inhibitors. The protein concentration of each sample was measured by bicinchoninic acid Kit (23227; Thermo Fisher Scientific). The proteins were separated by polyacrylamide gel electrophoresis and transferred onto a polyvinylidene fluoride (PVDF) membrane. After blocking with 5% bovine serum albumin for 1 hour, the PVDF membrane was incubated with the primary antibody to CXXC4 (1:1000, ab105400), ELK‐1 (1:500, ab32106), p‐ELK1 (ab28818, 1:100), CDK18 (1:3000, ab154557), ERK1/2 (1:1000, ab17942), p‐ERK1/2 (1:400, ab223500) and β‐actin (1:5000, ab179467) at 4°C overnight. On the next day, the membrane was incubated with horseradish peroxidase‐conjugated goat anti‐rabbit antibody to IgG (1:20 000, ab205718) at room temperature for 1.5 hour. All the above antibodies were purchased from Abcam Inc. Then, the results were visualized by adding developer (NCI4106; Pierce; Thermo Fisher Scientific). Image J 1.48u software (Bio‐Rad Laboratories) was used for protein quantitative analysis. The relative expression of protein was expressed as the ratio of gray value of protein band to be tested to that of internal reference band (β‐actin).

### 2.14 Xenograft tumour in nude mice

Female NOD/SCID mice (6‐8 weeks, 19‐22 g) were randomly divided into three groups, 10 per group. A total of 5 × 10^5^ SGC7901 cells treated with the sh‐CXXC4 or GDC‐0994 plasmids were injected into female NOD/SCID venules. After 2 weeks, the tumour size was measured with a Vernier caliper every 2 days. The tumour volume was calculated from three vertical measurements. The mice were killed using Pentobarbital sodium (50 mg/kg) (57‐33‐0; Shanghai Beizhuo Biotechnology Co., Ltd.), and then, the tumour was resected and photographed. The spleen was prepared into a single cell suspension for flow cytometry.^19^

### 2.15 Statistical analysis

SPSS 21.0 statistical software (IBM Corp.) was employed for statistical analysis. Measurement data were expressed as mean ± SD. The data conforming to normal distribution and homogeneous variance between two groups were analysed by paired (for paired data) or unpaired *t* test (for unpaired data). Comparisons among multiple groups were analysed using the one‐way analysis of variance (ANOVA) with Tukey's post hoc test used. The data at different time points were analysed by the repeated measures ANOVA, followed by Bonferroni's post hoc test. A value of *P* < .05 was considered as statistically significant.

## 3 RESULTS

### 3.1 CXXC4 overexpression curbed the proliferative potential of gastric cancer cells and promoted the activation of T cells

**Figure 1:**
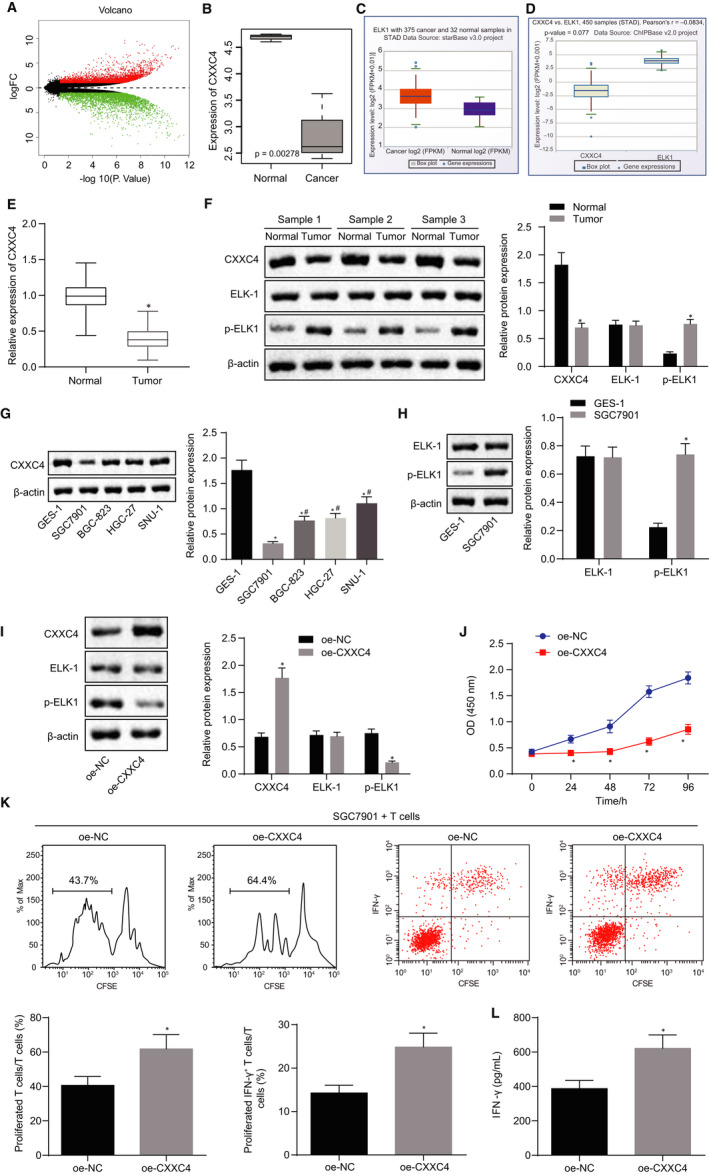
Overexpressed CXXC4 inhibits the proliferation of gastric cancer cells and promotes the activation of T cells by suppressing the phosphorylation of ELK1. A, Volcano map, the *x*‐axis represents the different log10 *P* value, and the *y*‐axis indicates logFoldChange. Each point on the map represents a gene; red colour represents up‐regulated genes, and green refers to down‐regulated genes in gastric cancer samples. B, The expression box plot of CXXC4 in the gastric cancer microarray dataset GSE49051. The *x*‐axis represents the sample type, while the *y*‐axis represents the expression value. C, The expression box plot of ELK1 in gastric cancer in the Starbase database. D, The co‐expression box plot of CXXC4 and ELK1 according to Chipbase E, The expression of CXXC4 in gastric cancer and adjacent normal tissues measured by RT‐qPCR, n = 37. *P < .05 tumour tissues vs adjacent normal tissues. F, The expression of CXXC4, ELK‐1 and p‐ELK1 in gastric cancer and adjacent normal tissues normalized to β‐actin assessed by Western blot analysis, n = 37, *P < .05 tumour tissues *vs*. adjacent normal tissues. G, The expression of CXXC4 in normal gastric epithelial cell lines GES‐1 and gastric cancer cell lines SGC7901, BGC‐823, SNU‐1, HGC‐27 normalized to β‐actin measured by Western blot analysis, n = 3, *P < .05 SGC7901, BGC‐823, SNU‐1 or HGC‐27 cell line vs GES‐1 cell line, ^#^ *P* < .05 BGC‐823, SNU‐1 or HGC‐27 cell line vs SGC7901 cell line. H, The expression of ELK‐1 and p‐ELK1 in GES‐1 and the gastric cancer cell line SGC7901 normalized to β‐actin and assessed by Western blot, n = 3, *P < .05 SGC7901, BGC‐823, SNU‐1 or HGC‐27 cell line vs GES‐1 cell line, ^#^ *P* < .05 BGC‐823, SNU‐1 or HGC‐27 cell line vs SGC7901 cell line. I, The expression of CXXC4, ELK‐1 and p‐ELK1 in SGC7901 cells normalized to β‐actin after transfection with oe‐NC and oe‐CXXC4 measured by Western blot analysis, n = 3, *P < .05 oe‐CXXC4 vs oe‐NC. J, The proliferation of SGC7901 cells detected by CCK‐8 assay, n = 3, *P < .05 oe‐CXXC4 vs oe‐NC. K, The proliferation of CD3+ T cells and the proportion of activated IFN‐γ+ T cells assessed by flow cytometry, n = 3, *P < .05 oe‐CXXC4 vs oe‐NC. L, The cytokine IFN‐γ secreted by T cells detected by ELISA, n = 3, *P < .05 oe‐CXXC4 vs oe‐NC. Measurement data were expressed as mean ± SD. The data conforming to normal distribution and homogeneous variance between two groups were analysed by paired (for paired data in panels E and F) or unpaired *t* test (for unpaired data in panels H‐L). Comparisons in panel G among multiple groups were analysed using one‐way ANOVA, followed by Tukey's post hoc test. The data at different time points in panel J were analysed by the repeated measures ANOVA followed by Bonferroni's post hoc test. The experiment was repeated three times independently

In an attempt to investigate the effects of CXXC4 on the development of gastric cancer, the differential analysis on the gastric cancer microarray dataset GSE49051 was performed; 6924 DEGs were obtained, of which 3449 genes were highly expressed with the remaining 3475 genes being poorly expressed (Figure 1A). Subsequently, differential analysis of the microarray dataset GSE49051 showed that CXXC4 was expressed at a low level in gastric cancer (Figure 1B). The StarBase database revealed that ELK1 was highly expressed in gastric cancer (Figure 1C). The co‐expression relationship between CXXC4 and ELK1 in gastric cancer was obtained from Chipbase (Figure 1D). Then, RT‐qPCR revealed that the expression of CXXC4 in gastric cancer tissues was notably decreased compared to that of adjacent normal tissues (Figure 1E). According to Western blot analysis, compared with adjacent normal tissues, the expression of CXXC4 in gastric cancer declined while the level of p‐ELK1 increased (Figure 1F). Then, the expression of CXXC4 in normal gastric epithelial cell lines, GES‐1, and gastric cancer cell lines SGC7901, BGC‐823, SNU‐1 and HGC‐27 was assessed by Western blot. The results showed that CXXC4 was expressed at a lower level in gastric cancer cell lines compared to the GES‐1 cell line. Furthermore, SGC7901 having the lowest CXXC4 expression was selected for subsequent experiments (Figure 1G). Next, we measured the protein expression of ELK‐1 and p‐ELK1 in GES‐1 and SGC7901 cells, and found that the p‐ELK1 level was remarkably increased in SGC7901 cells compared to GES‐1 cells (Figure 1H).

In order to understand the effect of CXXC4 on the proliferative potential and immune escape capability of gastric cancer cells, we overexpressed CXXC4 in SGC7901 cells. As detected by Western blot analysis, the phosphorylation level of ELK1 decreased after overexpression of CXXC4 (Figure 1I). Moreover, the proliferation of SGC7901 cells measured by CCK‐8 assay revealed that the proliferative ability of SGC7901 cells was greatly decreased after overexpression of CXXC4 (Figure 1J). Then, as assessed by flow cytometry (Figure 1K), the number of proliferative CD3+ T cells and the proportion of IFN‐γ+ T cells were increased after being transfected with oe‐CXXC4, compared with the cells treated with oe‐NC. ELISA data showed that the cytokine IFN‐γ secreted by CD3+ T cells after transfection with oe‐CXXC4 was notably increased compared with cells treated with oe‐NC alone (Figure 1L). The above data suggest that overexpression of CXXC4 inhibited the proliferation of gastric cancer cells and promoted the activation of T cells by suppressing the phosphorylation of ELK1.

### 3.2 CXXC4 suppressed the expression of MIR100HG in gastric cancer cells

**Figure 2:**
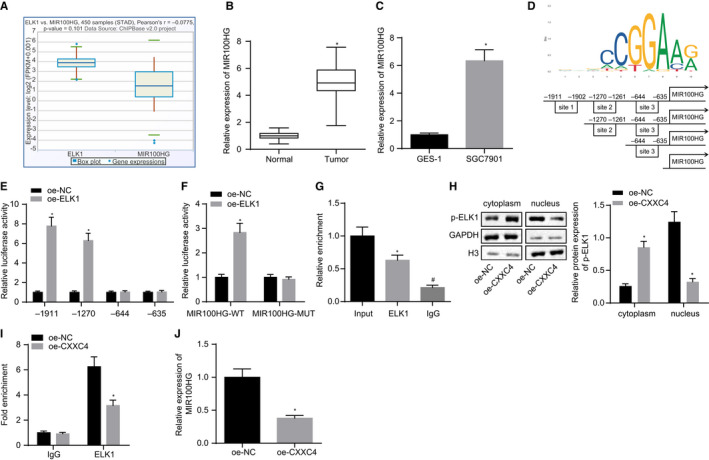
CXXC4 down‐regulates the expression of MIR100HG by inhibiting the phosphorylation of ELK1. A, The co‐expression box plot of ELK1 and MIR100HG of gastric cancer according to Chipbase. B, The expression of MIR100HG in gastric cancer tissues and adjacent normal tissues measured by RT‐qPCR, n = 37, *P < .05 tumour tissues vs adjacent normal tissues. C, The expression of MIR100HG in GES‐1 and the gastric cancer cell line SGC7901 assessed by RT‐qPCR, n = 3, *P < .05 SGC7901, BGC‐823, SNU‐1 or HGC‐27 cell line vs GES‐1 cell line. D, The binding sites predicted by JASPAR, and plasmids constructed by truncating putative binding sites. E, The binding site of ELK1 in MIR100HG promoter region detected by a dual‐luciferase reporter gene assay, n = 3, *P < .05 oe‐CXXC4 vs oe‐NC. F, The binding site mutation further confirmed by dual‐luciferase reporter gene assay, n = 3, *P < .05 oe‐CXXC4 vs oe‐NC. G, The combination of p‐ELK1 and MIR100HG promoter measured by ChIP assay, n = 3, * *P* < .05 ELK1 vs Input, ^#^ *P* < .05 IgG vs ELK1. H, The distribution of p‐ELK1 in cells detected by subcellular fraction and Western blot analysis, n = 3, *P < .05 oe‐CXXC4 vs oe‐NC. I, The combination of ELK1 and MIR100HG promoter assessed by ChIP assay, n = 3, *P < .05 oe‐CXXC4 vs oe‐NC. J, The expression of MIR100HG measured by RT‐qPCR, n = 3, * *P* < .05 oe‐CXXC4 vs oe‐NC. Measurement data were expressed as mean ± SD. Comparisons between tumour tissues and adjacent normal tissues in panel B were analysed using paired *t* test. The data conforming to normal distribution and homogeneous variance between two groups in panels C and E‐J were analysed by unpaired *t* test. Comparisons among multiple groups in panel G were analysed using one‐way ANOVA, followed by Tukey's post hoc test. The experiment was repeated three times independently

To further study the mechanism of action for ELK1/MIR100HG in gastric cancer, the co‐expression relationship between ELK1 and MIR100HG was retrieved and confirmed using the Chipbase website (Figure 2A). The expression of MIR100HG in gastric cancer was measured by RT‐qPCR, and the results showed that compared with normal tissues, the expression of MIR100HG was increased in gastric cancer tissues (Figure 2B). Cell lines’ data also showed a similar trend (Figure 2C). The binding sites of ELK1 in the MIR100HG promoter region were predicted by JASPAR, and all three predicted binding sites were truncated (Figure 2D). A dual‐luciferase reporter gene assay found that the possible binding site of ELK1 in the MIR100HG promoter region was site 2 (Figure 2E), which was mutated further. Then, ELK1 was found to improve the luciferase activity of wild‐type MIR100HG, but had no significant effect on the mutant (Figure 2F), indicating that ELK1 might bind to the site 2 of the MIR100HG promoter. ChIP assay revealed the combination of p‐ELK1 and MIR100HG promoter (Figure 2G). As a transcription factor, ELK1 regulates downstream genes by conducting nuclear import, an event triggered by ELK1 phosphorylation.^25^ Therefore, we speculated that CXXC4 might inhibit the nuclear import of ELK1 by suppressing the phosphorylation of ELK1, and then inhibiting the expression of MIR100HG. Therefore, after overexpression of CXXC4 in SGC7901 cells, the distribution of p‐ELK1 in the cells was detected by subcellular fraction. The results showed that phosphorylated ELK1 was translocated into the nucleus, while overexpression of CXXC4 inhibited the phosphorylation of ELK1 and thus suppressed the nuclear import of ELK1 (Figure 2H). ChIP assay further suggested that the binding of the ELK1 to the promoter of MIR100HG decreased (Figure 2I), and the expression of MIR100HG declined notably after overexpression of CXXC4 (Figure 2H). Taken together, our data demonstrated that nuclear translocation of ELK1 was inhibited by CXXC4 through suppression on the extent of ELK1 phosphorylation.

### 3.3 CXXC4 overexpression inhibited the proliferation of gastric cancer cells and promoted the activation of T cells by down‐regulating the expression of MIR100HG

**Figure 3:**
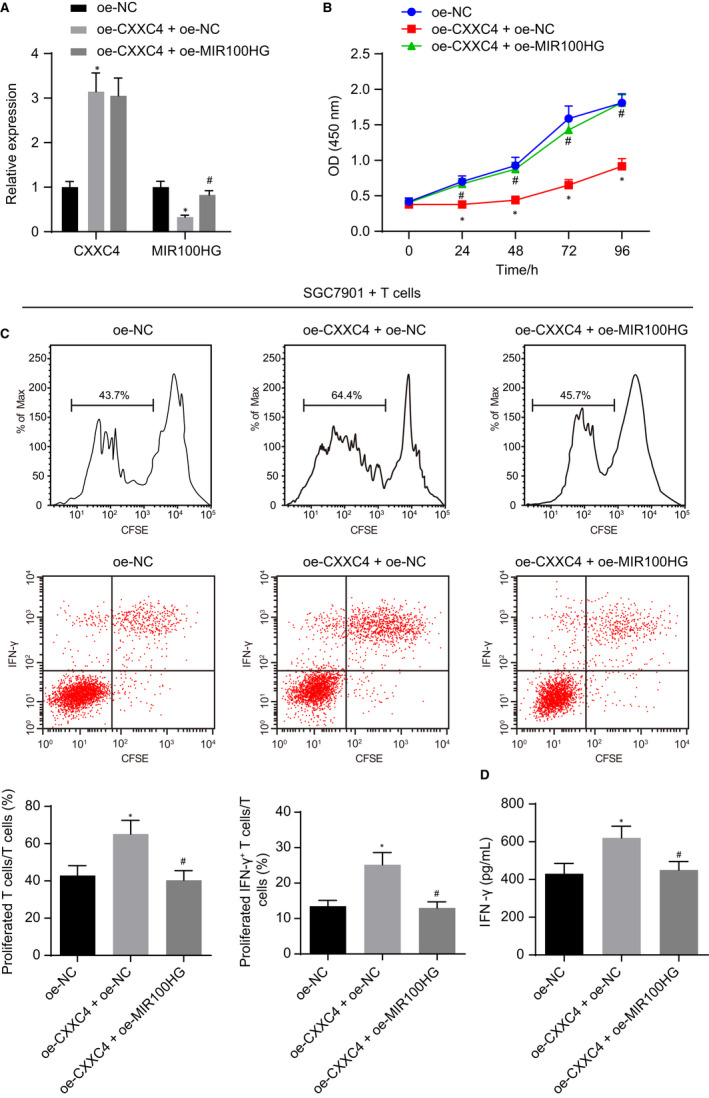
Up‐regulated CXXC4 exerts inhibitory effects on the proliferation of gastric cancer cells and activation of T cells by inhibiting the expression of MIR100HG. A, The expression of CXXC4 and MIR100HG measured by RT‐qPCR, n = 3. B, The proliferation of SGC7901 cells detected by CCK‐8 assay, n = 3. C, The proliferative ability of CD3+ T cell and the proportion of activated IFN‐γ+ T cells detected by flow cytometry, n = 3. D, The IFN‐γ cytokine secreted by T cells measured by ELISA, n = 3, *P < .05 oe‐CXXC4 + oe‐NC vs oe‐NC, ^#^ *P* < .05 oe‐CXXC4 + oe‐MIR100HG vs oe‐CXXC4 + oe‐NC. Measurement data were expressed as mean ± SD. Comparisons between two groups in panels A, C and D were analysed by unpaired *t* test. Comparisons among multiple groups in panels A, C and D were analysed using one‐way ANOVA, followed by Bonferroni's post hoc test. The data at different time points in panel B were analysed by the repeated measures ANOVA followed by Bonferroni's post hoc test. The experiment was repeated three times independently

The aforementioned results have determined that highly expressed CXXC4 can inhibit the proliferation of gastric cancer cells and promote the activation of T cells, and CXXC4 can inhibit the expression of MIR100HG by inhibiting the phosphorylation of ELK1. Therefore, we speculated that the regulation of CXXC4 on the proliferation and immune escape potential of gastric cancer cells may be realized by MIR100HG. We overexpressed CXXC4 in SGC7901 cells, and RT‐qPCR revealed that the expression of CXXC4 was significantly increased, as expected, while the expression of MIR100HG decreased significantly in response to CXXC4 overexpression. Furthermore, simultaneously overexpressing CXXC4 and MIR100HG reversed the inhibition of MIR100HG expression induced by overexpression of CXXC4 alone (Figure 3A). As detected by a CCK‐8 assay, overexpression of CXXC4 reduced the proliferation of SGC7901, while additional overexpression of MIR100HG counterweighed the inhibition on the proliferation of SGC7901 induced by overexpressing CXXC4 (Figure 3B). Moreover, the proliferation of CD3+ T cells and the proportion of IFN‐γ+ T cells were found to be elevated after overexpressing CXXC4 (Figure 3C). Meanwhile, the results of an ELISA assay showed that the IFN‐γ cytokine secreted by CD3+ T cells increased after overexpression of CXXC4 (Figure 3D), while simultaneously overexpressing CXXC4 and MIR100HG could abolish the effect of CXXC4 on T cells. These results indicated that overexpression of CXXC4 could suppress the proliferation of gastric cancer cells and promote the activation of T cells by down‐regulating MIR100HG.

### 3.4 CXXC4 overexpression curbed the proliferation of gastric cancer cells and enhanced the activation of T cells by suppressing the expression of CDK18

**Figure 4:**
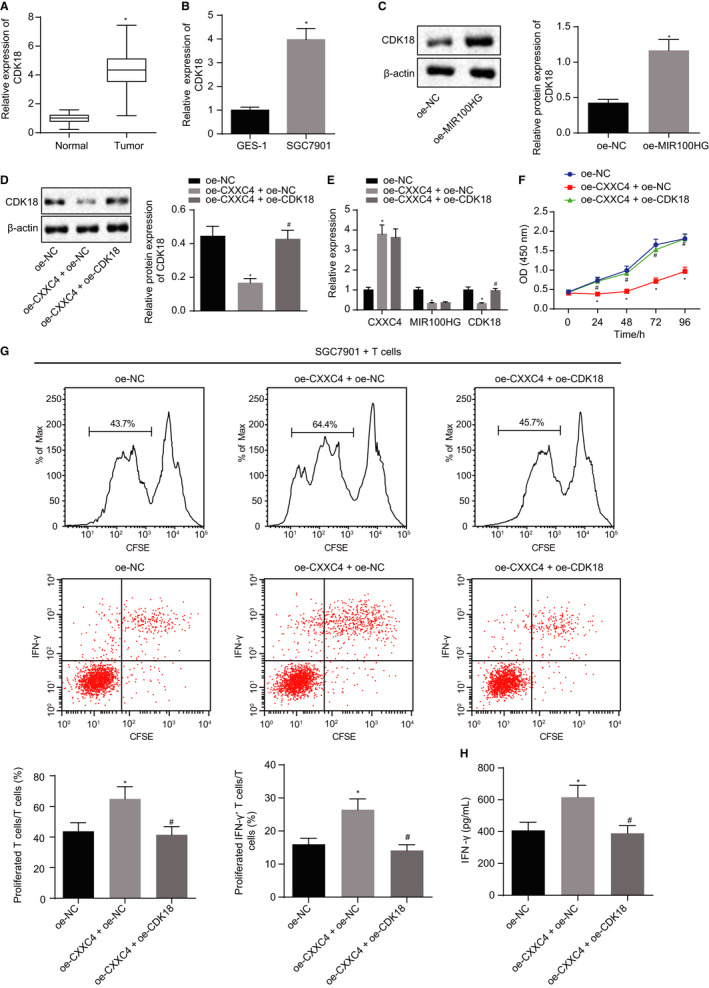
Enforced expression of CXXC4 leads to suppression of gastric cancer cells proliferation and the activation of T cells by inhibiting the expression of CDK18. A, The expression of CDK18 in gastric cancer and adjacent normal tissues measured by RT‐qPCR, n = 37, *P < .05 tumour tissues vs adjacent normal tissues. B, The expression of CDK18 in GES‐1 and the gastric cancer cell line SGC7901 assessed by RT‐qPCR, n = 3, *P < .05 SGC7091 cell line vs GES‐1 cell line. C, The expression of CDK18 in gastric cancer cell line SGC7901 normalized to β‐actin measured by Western blot analysis, n = 3, *P < .05 oe‐MIR100HG vs oe‐NC. D, The expression of CDK18 in gastric cancer cell line SGC7901 normalized to β‐actin assessed by Western blot analysis, n = 3. E, The expression of CXXC4, MIR100HG and CDK18 measured by RT‐qPCR, n = 3. F, The proliferation of SGC7901 cells detected by CCK‐8 assay, n = 3. G, The proliferation ability and the proportion of activated IFN‐γ+ T cells of CD3+ detected by flow cytometry. H, The cytokine IFN‐γ secreted by T cells measured by ELISA, n = 3, *P < .05 oe‐CXXC4 + oe‐NC vs oe‐NC, ^#^ *P* < .05 oe‐CXXC4 + oe‐CDK18 vs oe‐CXXC4 + oe‐NC. Measurement data were expressed as mean ± SD. Comparisons between tumour and adjacent normal tissues in panel A were analysed using paired *t* test. The data conforming to normal distribution and homogeneous variance between two groups in panels B and C were analysed by unpaired *t* test. Comparisons among multiple groups in panels D, E, G and H were analysed using the one‐way ANOVA, followed by Tukey's post hoc test. The data at different time points in panel F were analysed by the repeated measures ANOVA followed by Bonferroni's post hoc test. The experiment was repeated three times independently

With an attempt to further explore the correlation between CDK18 and gastric cancer, the expression of CDK18 in gastric cancer tissues and cells was measured, and the results showed that the expression of CDK18 was increased in gastric cancer tissues and cells (Figure 4A,B). After overexpressing MIR100HG in SGC7901 cells, an elevated expression of CDK18 was observed (Figure 4C). As revealed from Western blot analysis in the presence of up‐regulated MIR100HG, overexpression of CXXC4 reduced the expression of CDK18, while overexpression of CXXC4 and MIR100HG counteracted the action of CXXC4 overexpression alone (Figure 4D). These findings suggest that CXXC4 could inhibit the expression of CDK18 via MIR100HG in gastric cancer cells.

To further study whether CDK18 is instrumental in the regulatory role of CXXC4 on the proliferation and immune escape of gastric cancer cells, CXXC4 and CDK18 were overexpressed in SGC7901 cells. First, RT‐qPCR showed that overexpression of CXXC4 could inhibit the expression of CDK18, while additional overexpression of CDK18 reversed these results (Figure 4E). As detected by a CCK‐8 assay, suppressed proliferation of SGC7901 induced by overexpression of CXXC4 was reversed by further treatment with oe‐CDK18 (Figure 4F). We then co‐cultured the transfected gastric cancer cells with CD3+ T cells, followed by flow cytometric analysis (Figure 4G), results of which indicated that the number of proliferative CD3+ T cells and the proportion of IFN‐γ+ T cells increased remarkably after overexpression of CXXC4. The results of an ELISA also showed that IFN‐γ secreted by CD3+ T cells notably elevated after overexpression of CXXC4 (Figure 4H), while simultaneously overexpressing CXXC4 and CDK18 abrogated the action of overexpressing CXXC4 on T cells. The above results showed that overexpressed CXXC4 suppressed the proliferation of gastric cancer cells and promoted the activation of T cells by down‐regulating CDK18 through MIR100HG.

### 3.5 CXXC4/CDK18‐ERK1/2 axis contributed to gastric cancer progression through MIR100HG

**Figure 5:**
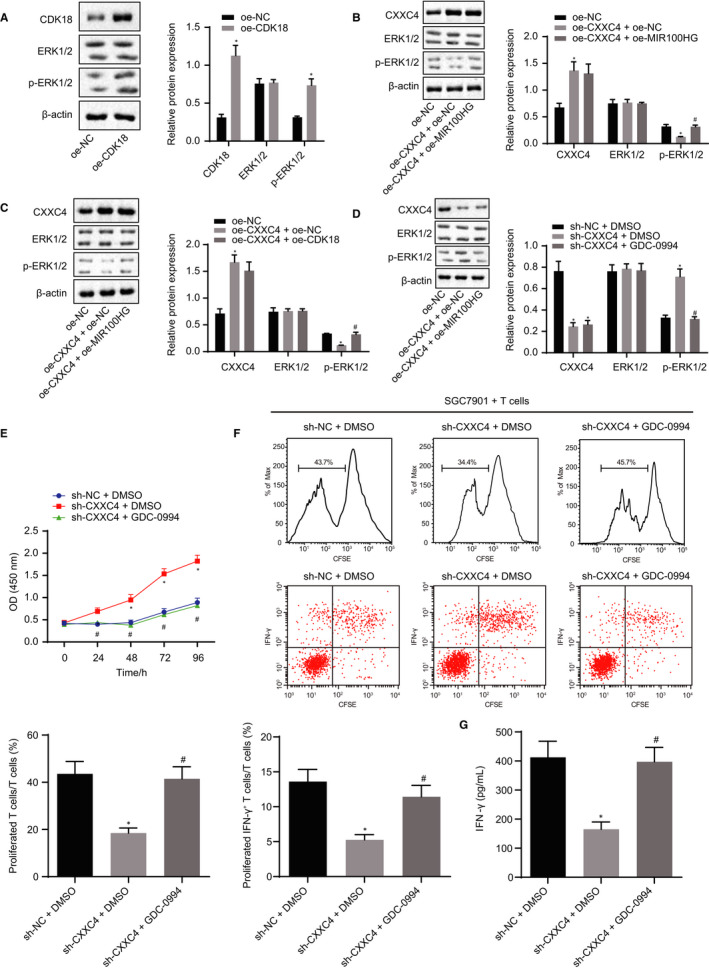
Re‐expression of CXXC4 suppresses the proliferation of gastric cancer cells and promotes the activation of T cells by inhibiting CDK18‐ERK1/2 axis through MIR100HG. A, The expression of CDK18, ERK1/2 and p‐ERK1/2 normalized to β‐actin measured by Western blot analysis in SGC7901 cells, n = 3, *P < .05 oe‐CDK18 vs oe‐NC. B, The expression of ERK1/2 and p‐ERK1/2 normalized to β‐actin assessed by Western blot analysis in SGC7901 cells, n = 3, *P < .05 oe‐CXXC4 + oe‐NC vs oe‐NC, ^#^ *P* < .05 oe‐CXXC4 + oe‐MIR100HG vs oe‐CXXC4 + oe‐NC. (C), The expression of ERK1/2 and p‐ERK1/2 normalized to β‐actin measured by Western blot analysis, n = 3. *P < .05 oe‐CXXC4 + oe‐NC vs oe‐NC, ^#^ *P* < .05 oe‐CXXC4 + oe‐CDK18 vs oe‐CXXC4 + oe‐NC. D, The expression of CXXC4, ERK1/2 and p‐ERK1/2 normalized to β‐actin assessed by Western blot analysis in SGC7901 cells, n = 3. E, The proliferation of SGC7901 gastric cancer cells detected by CCK‐8, n = 3. F, The proliferation of CD3+ T cells and the proportion of IFN‐γ+ T cells measured by flow cytometry, n = 3. G, The IFN‐γ cytokine secreted by T cells assessed by ELISA, n = 3. *P < .05 sh‐CXXC4 + DMSO vs sh‐NC + DMSO, ^#^ *P* < .05 sh‐CXXC4 + GDC‐0994 vs sh‐CXXC4 + DMSO. Statistical data were measurement data and presented as mean ± SD. Unpaired student's *t* test was used for comparison between the two groups in panel A, and comparisons among multiple groups were analysed by the one‐way ANOVA in panels B‐D, F and G. The data at different time points in panel E were analysed by the repeated measures ANOVA, followed by Bonferroni's post hoc test. The experiment was repeated three times independently

Next, we verified the role of the CXXC4/MIR100HG/CDK18‐ERK1/2 axis in gastric cancer. As revealed by a Western blot assay, the expression of CDK18 and the extent of ERK1/2 phosphorylation increased after overexpressing CDK18 (Figure 5A). Furthermore, overexpression of CXXC4 declined the extent of ERK1/2 phosphorylation, while further overexpression of MIR100HG or CDK18 reversed the effect of overexpressing CXXC4 (Figure 5B,C). Subsequently, we silenced CXXC4 and inhibited ERK1/2 axis in SGC7901 cells. After silencing CXXC4, the extent of ERK1/2 phosphorylation was elevated (Figure 5D), and the proliferation ability of SGC7901 cells was also significantly increased (Figure 5E). In contrast, the proliferation of CD3+ T cells and the proportion of IFN‐γ+ T cells were notably reduced (Figure 5F) and the cytokine IFN‐γ secreted by the CD3+ T cells declined (Figure 5G). However, the inhibition of ERK1/2 axis would counterbalance the action of silencing CXXC4 alone. These results suggest that highly expressed CXXC4 could inhibit the CDK18‐ERK1/2 axis through MIR100HG, thus suppressing the proliferation of gastric cancer cells and promoting the activation of T cells.

### 3.6 In vivo inhibitory role of overexpressed CXXC4 in immune escape of gastric cancer cells through the ERK1/2 axis

**Figure 6:**
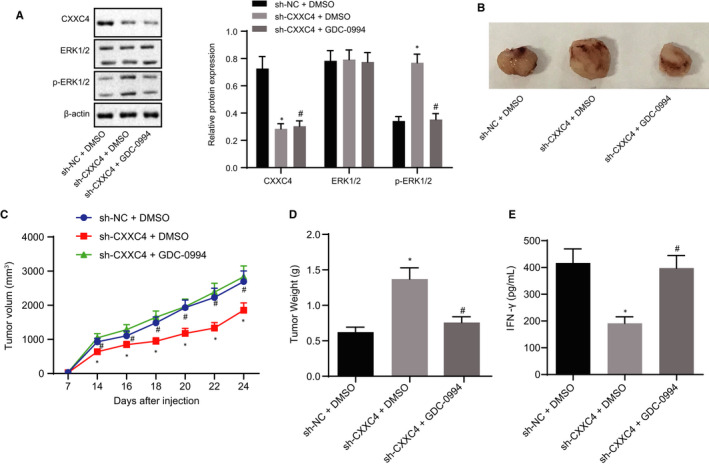
Immune escape of gastric cancer cells is suppressed by the CXXC4/ERK1/2 axis in vivo. A, The expression of CXXC4, ERK1/2 and p‐ERK1/2 normalized to β‐actin assessed by Western blot analysis, n = 5. B, Representative images of resected tumours from mice, n = 10. C, Tumour growth curve of mice in each group, n = 10. D, Tumour weight of mice in each group, n = 10. E, The expression of IFN‐γ in spleen cells lysate detected by ELISA, n = 10, *P < .05 sh‐CXXC4 + DMSO vs sh‐NC + DMSO, ^#^ *P* < .05 sh‐CXXC4 + GDC‐0994 vs sh‐CXXC4 + DMSO. Statistical data were measurement data and presented as mean ± SD. Comparisons among multiple groups in panels A, D and E were analysed by the one‐way ANOVA. The data at different time points in panel C were analysed by the repeated measures ANOVA, followed by Bonferroni's post hoc test. The experiment was repeated three times independently

With an attempt to further validate our results in vivo, we established a tumour model of nude mice. Western blot analysis illustrated that silencing CXXC4 resulted in elevated extent of ERK1/2 phosphorylation and accelerated tumour growth, and increased volume and weight of tumours in mice (Figure 6A‐D). The expression of IFN‐γ in the lysate of spleen cells was assessed by ELISA. The results revealed that the cytokine IFN‐γ secreted by the cells decreased after silencing CXXC4 (Figure 6E). Also, the results were reversed by further inhibition of the ERK1/2 axis. To sum up, the CXXC4/CDK18‐ERK1/2 axis was verified to be functional in vivo.

## 4 DISCUSSION

Tumour‐infiltrating lymphocytes (TILs) are commonly observed in tumour tissues and compete with tumour cells to grab nutritive materials in microenvironment.^26^ Additionally, TILs are the major immune cells to defend tumour cells. TILs are composed of multiple types of lymphocytes in which CD8+ T cells are the effectors which clear cancer cells.^27^ A higher proportion of CD8+ T cells indicates a higher potential of adaptive immune resistance. Thus, the study of CD8+ T cell activation in tumour tissues presents high value to acknowledge the immunotherapy of cancer. Here, we reported that tumour suppressor protein CXXC4 was able to improve proliferation of CD3+ T cells through ELK1‐mediated regulation of ERK1/2 axis, resulting in an enhanced immune effect to gastric cancer cells.

First, we found highly expressed CXXC4 could inhibit the proliferation of gastric cancer cells and enhance the activation of T cells through phosphorylation of ELK1. CXXC4 is a CpG‐binding protein which is involved with virus related gastric cancer through regulation of DNA methylation.^28^ Besides epigenetic regulation, CXXC4 can also promote cellular apoptosis via up‐regulation of growth and differentiation factor 15 in gastric cancer.^7^ Previous studies along with our results confirm that CXXC4 may function as a tumour suppressor in gastric cancer. Although few reports have presented the regulation of T cell destiny by CXXC4, ELK1 has been shown to regulate lymphocyte fate. For example, ERK/ELK1‐mediated BTB and CNC homolog 2 (Bach1) repression can control the differentiation of human naïve B cells.^28^ Innate‐like CD8+ T cell differentiation can be promoted by low‐level ERK signalling through ELK1 and ELK4.^11^ Phosphorylation of ERK indicates the activation of the MAPK axis, which can further phosphorylate the EKL1 protein. The ERK phosphorylation cascade mediates promotion of proliferation and differentiation.^29^ ELK1, containing multiple copies of the MAPK core consensus motif, can be stoichiometrically phosphorylated at its C terminal domain after stimulation of proliferation.^30^ However, how SGC7901 cells overexpressing CXXC4 transduced signalling and promoted T cell growth should be further investigated.

Second, we demonstrated that CXXC4 could inhibit the CDK18‐ERK1/2 axis through MIR100HG. Phosphorylation of ELK1 proteins promotes its translocation to the cellular nucleus.^25^ We found that low level of phosphorylation of ELK1 proteins inhibited transcription of MIR100HG, one oncogenic lncRNA. MIR100HG contains two microRNA (miRNA) gene locus, miR‐100 and miR‐125. Up‐regulation of MIR100HG results in overexpression of miR‐100 and miR‐125, which are important negative regulators of five Wnt/beta‐catenin.^12^ We inferred that regulation of the CDK18‐ERK1/2 axis by MIR100HG was similarly mediated by some miRNAs. Phosphorylation of ERK1/2 represents strong signals of T cell proliferation. For instance, Galectin‐8 triggers significant phosphorylation of ZAP70 and ERK1/2, enhancing independent T cell proliferation.^12^ ERK is not only important for T cell function, but this axis is also critical for other immune cells such as dendritic cells, natural killer T cell, T helper cell and regulatory T cell.^31^, ^32^

In this research, the functions of CXXC4 in regulating the fate of tumour cells and differentiation at the T cell level were intriguing. We elucidated one signalling axis from CXXC4, ELK1, MIR100HG to ERK1/2. However, previous researches have shown that the ELK1 protein is a downstream target of ERK1/2, activated ERK efficiently phosphorylating nuclear ELK1.^33^, ^34^ Another study shows reciprocal regulation of the transcription activation, and repressive activities are collaborated by MAPK‐mediated phosphorylation of ELK1.^35^ Given these results, we speculated that ELK1 and ERK1/2 might form a circular‐feedback regulation mechanism. However, there are still lots of work to do to explore the involving participant molecules as many as possible to better understand the mechanism. Of note, only NCs were set in our investigation due to the limited conditions, and further studies should be performed by including internal positive controls for validation of our results. Additional attention should also be paid when relating the findings from animal models to the clinical setting because of physiological and pathological differences. Collectively, CXXC4 is a tumour suppressor that controls the growth of tumour cells and the statuses of T cells which will further target the tumour cells and kill them. MIR100HG and ERK axis can mediate the immune system activity and hence should be considered as chemical or antibody target for immunotherapy in the future.

## 5 CONCLUSION

CXXC4 overexpression has been shown to exert inhibitory effects on immune escape of gastric cancer cells, by suppressing SGC7901 cell proliferation and promoting release of IFN‐γ by CD3+ T cells via the inactivated CDK18‐ERK1/2 signalling pathway through down‐regulation of ELK1 phosphorylation and MIR100HG expression.

## CONFLICT OF INTEREST

The authors declare that they have no competing interests.

## AUTHOR CONTRIBUTIONS

**Ping Li:** Conceptualization (equal); Investigation (equal). **Dongfang Ge:** Resources (equal); Writing‐review & editing (equal). **Pengfei Li:** Project administration (equal); Visualization (equal). **Fangyong Hu:** Conceptualization (equal); Validation (equal). **Junfeng Chu:** Data curation (equal); Software (equal). **Xiaojun Chen:** Investigation (equal); Software (equal). **Wenbo Song:** Data curation (equal); Resources (equal). **Ali Wang:** Formal analysis (equal); Validation (equal). **Guangyu Tian:** Methodology (equal); Writing‐review & editing (equal). **Xiang Gu:** Writing‐original draft (equal).

## Data Availability

Research data not shared.
